# Cost Analysis of Accidents according to Demographic Factors in Iran

**Published:** 2019-07

**Authors:** Reza HASHEMPOUR, Ali TAHMASEBI, Mohammad VEYSI, Milad AMINI, Nader TAVAKOLI

**Affiliations:** 1. Department of Health Management and Economics, School of Public Health, Tehran University of Medical Sciences, Tehran, Iran; 2. Trauma and Injury Research Center, Iran University of Medical Sciences, Tehran, Iran; 3. Health Management and Economics Research Center, Iran University of Medical Sciences, Tehran, Iran

**Keywords:** Epidemiology, Cost analysis, Accidents, Iran

## Abstract

**Background:**

The first cause of death and disability constituting to the burden of disease in Iran has been accident and injury incidents. Young people are more at risk, these accidents have a negative effect on the national gross domestic product (GDP), on the one hand, and they increase the costs of the health system on the other hand. Therefore, this study aimed to analyze the costs and epidemiological pattern of accidents.

**Methods:**

The study variables in the first 8 months of 2016 included from Tehran Province, Iran; age, sex, how to transfer to the hospital, consequences of an accident, the injured area, and costs. Overall, 400 cases were investigated by referring to the patients' files and deriving the intended data. SPSS software used to analyze the data and statistical tests of t-test and ANOVA were applied.

**Results:**

Most accidents have happened in summer months of Jun, Jul and Aug. Most of the files (48%) were related to the age group of 16–30. The mean cost was 9024.82 dollar. In total, 39% of the road accidents had occurred by motorcycle and 90.8% of the patients discharged from the hospital.

**Conclusion:**

Since about half of the traffic accidents were related to the age of 16 to 30 who were the active population of a community, therefore, any disability or death in these groups could cause an economic burden on the community and increase DALY. Thus, it appears essential to develop proper programs such as education for appropriate driving and strict rules for giving driving license to these ages.

## Introduction

Although vehicles have created relative prosperity for human beings, it has brought a new problem which is road traffic accident. Nowadays, road traffic accident is considered as one of the three risk factors in terms of economic, social and health grounds throughout the world (1) and Road Accident Injuries (RAIs) indicator have been a public health dilemma all over the world (2).

Injuries have resulted in almost five million deaths and 25–50 million disabilities each year which comprises 16% of the total burden of disease. More than 90% of these mortality and disability rates occur in middle-income and low-income countries, bearing the fact that they have almost 54% of the world’s vehicle and their injury control system has been weak (3–4) and if no special action is taken, accidents will be the seventh cause of death by 2030 (5). Cost of their services is more than 160 billion dollars per year. Almost 60% of the deaths were caused by traffic accidents occurring among people aged 15 to 44 and it is unfortunate that this mortality has been rising (6).

There is a need to spend a great amount of expenditure on the costs for hospital treatments including trauma care. Direct and indirect costs, as well as productivity losses, have had a range of 1.3% to 3% of GDP loss/impact (7). Incidence rate of traffic accidents in Iran has been 26.5 in 100.000 people per year (8). Noting that middle-income countries have the highest mortality rate caused by road traffic accidents (20.1 in 100.000) (9), vital importance to reduce mortality rate from the accidents in these countries so that total burden of the mortality rate reduces as well (9).

Poor people in low- and middle-income countries are more exposed to traumas showing an asymmetric distribution of injuries. In general, 90% of road traffic injuries have occurred in these environments and the burden had an increasing trend due to rapid growth of urban and motorization without improvements in vehicle safety design and road and traffic safety (10, 11). In Sep 2015, Sustainable Development Goals (SDGs) were presented worldwide by managers and experts including two relevant goals to road safety. First, SDG 3 (goal no. 3) indicated assurance of good health and promoting well-being in all ages, especially by reducing all deaths relating to traffic accident by half until 2020. Secondly, SDG 11 (goal no. 11) suggested making cities sustainable, resilient and safe for all (12).

In Tehran, the economic burden of road traffic accidents has been estimated to be 0.3% to 0.4% of GDP (1), while the total economic burden of road traffic accidents in Iran has been estimated to be 2.19% of GDP (13). RAIs caused economic losses of about 6 billion dollars per year in Iran (14) and in the scope the world, 16 thousand people die every day because of wounds and injuries (2)

The mean hospitalization cost for each patient was estimated to be 1622 dollars (15). Moreover, there were costs of traffic accidents even after discharge from hospital. There are 74.8% more costs for traffic accidents in a year after discharge, comparing to a year before the traffic accident (16). Many studies have been carried out about accidents in Iran but still, the costs of accidents are not separated based on demographic variables (17–21). Therefore, considering high costs relating to road accident injuries, appropriate strategies are required for the injuries as well as analysis and evaluation of the injuries from economic perspective which could be an important step in implementation of preventive programs. In this regard, we aimed to analyze the costs based on demographic variables in order to determine the groups at risk and their costs.

## Materials and Methods

A retrospective descriptive study was used and addressed residences in Tehran Province. The study population were injured traffic accident people transferred to Emergency Department by ambulances, by their own cars, by police and other ways between Mar and Nov 2016, in Tehran, Iran. The injured areas were divided into four categories: hand and foot, head, chest and abdomen and back, and other tissues that could be. The accident result was divided into four categories: discharging, death, discharge against medical advice, and others. Others might vary like transferring to another hospital and etcetera. The total cost is all hospital costs, including medicine, operating room, laboratory, hospital, etc. Tehran as the capital of Iran with estimated population of 12, million residences has three medical sciences universities including Tehran, Iran and Shahid Beheshti which cover 16, 46, 43 hospitals, respectively. As inclusion criteria, governmental/public hospitals and hospitals with more than 100 medical records were selected for our study; and nongovernmental/private hospitals and hospital with less than 100 medical records were excluded from the study. To maximized the sample size, the value of p and q was 0/5 and the value of d was 5% and Z_1-a / 2_ was 1/96. The sample size was 384. In the next step, the number of records for each university and hospital was selected randomly based on proportionate stratified random sampling. Overall, 45,525 cases were selected and the share of Tehran University of Medical Sciences, Iran and Shahid Beheshti were 6903, 21040, and 17582, respectively. Using proportionate stratified random sampling, the contribution of each university was estimated at 58, 178 and 148, respectively. The sampling was done for hospitals, and then the sample size for Tehran, Iran and Shahid Beheshti Medical Sciences Universities reached 63, 182 and 148, respectively. The data were collected through Center of Medical Documents assessment. The studied variables were: age; sex; the month of occurrence; university; hospital; transfer to hospital, region of injury; outcome; total cost.

Statistical analysis was done by “SPSS 20 software at descriptive and analytical levels.

## Results

The study comprised an investigation of 400 medical records of the RTIs patients. According to inclusion and exclusion criteria 182, 155, 63 medical records were selected through stratified sampling and evaluated for Iran, Shahid Beheshti and Tehran universities of medical science, respectively. Considering stratified sampling, medical record distribution for hospitals were obtained (Table 1) and no significant differences were revealed among hospitals and universities. 17.5 (%) patients were admitted to emergency department between 01:00 am and 06:00 am; 15.5 (%) between 07:00 am and 12:00 pm; 25 (%) between 13:00 and 18:00; and 33.8 (%) between 19 and 24:00; and 8.3% were missing. Statistical differences were revealed when costs of the injured men versus women were compared (Table 2). The studied patients were 309 (77.25%) male and 91 (22.75%) female with a mean age of 31.57±0.77 (range 1–94). High incidence was seen in the months of Aug (16.3%), Jun (15.8%), Jul (14.8%) and low numbers were in Mar (5.8%), Apr (9.8%) and Oct (11.5%) (Table 3). The costs of 400 patients in this study were evaluated and the mean cost was 9024.82 USD. Minimum and maximum were 38 USD and 529596 USD, respectively. The patient costs for each month are given in Table 3 and there is no significant difference among the months. The highest frequency for age was observed between 16 and 30 yr old (48%) patients and the mean costs had significance difference among 46–60 age group with the others exception >61 years old (Table 4). There were significant differences among patient outcomes and they were between Death and Discharge, DAMA (Discharge against Medical Advice) (Table 4).

**Table 1:** Distribution of accidents according to hospitals and universities

***University***	***Hospital***	***Number of medical records***	***P-value (among hospitals)***	***P-value (among universities)***
Iran University of Medical Sciences	Haft-e Tir	83	0.296	0.18
	Firouzgar	38		
	Rasoul-e-Akram	24		
	Hazrat-e-Fatemeh	20		
	Emam Sajad	15		
	Shafa Yahyaeian	2		
	Total	182		
Shahid Beheshti University of Medical Sciences	loqman	56	0.263	
	Emam Hosein	33		
	Akhtar	16		
	Shohadaye Tajrish	15		
	Taleqani	9		
	Sha‘ban	8		
	Mofatteh	5		
	Firouzkouh	5		
	Be‘sat	4		
	Panzdah-e khordad	2		
	Za‘eem pakdasht	2		
	Total	155		
Tehran University of Medical Sciences	Sina	20	0.849	
	Ziaeyan	11		
	Emam khomeini	10		
	Shari‘ati	9		
	Amir alam	6		
	Baharlou	4		
	Emam reza	3		
	Total	63		

**Table 2:** Distribution of the accident according to gender, mean cost and significance level

***Gender***	***Number***	***Percentage***	***Mean (USD)***	***P-value***
Male	309	77.25	10342.43	0.017
Female	91	22.75	4550.73	
Total	400	100	9024.82	

**Table 3:** Distribution of accidents according to months

***Month***	***Number(n)***	***Percentage***	***Mean (USD)***	***F***	***P-value***
March	23	5.8	22767	10572	0.142
April	39	9.8	7340		
May	50	12.5	4642		
June	63	15.8	4972		
July	59	14.8	2161		
August	65	16.3	6524		
September	55	13.8	5671		
October	45	11.5	19544		
Total	400	100	9024.82		

**Table 4:** Distribution of accident according to age and outcome

***Variable***	***Age (yr)***	***Number(n)***	***Percent***	***F***	***P-value***
Age group	<15	36	9	3.145	0.015
	16–30	192	48		
	31–45	99	24.8		
	46–60	51	12.8		
	>61	22	5.5		
	Total	400	100		
Outcome	Discharge	363	90.8	41.776	0.000
	Death	7	1.8		
	DAMA^*^	20	5.0		
	Other	10	2.5		

* Discharge against medical advice

According to the kind of vehicle in the patient records, 156 (39%) accidents associated with motorcycle accident. The results according to type of vehicle are given in Table 5. Mortality occurred in seven patients (1.8%) and no significant difference was found among the types of accident or vehicle (Table 5). The most frequent injury was observed in hand and foot trauma (47%), while other traumas were in head (27%), thorax and abdomen (8.3%) and others (17%).

Differences among the areas of injury were not observed (Table 5). The most frequent type of transfer to hospital was by ambulance and there was no significant difference among different types of transfer with respect to cost of care (Table 5).

**Table 5:** Distribution of accidents according to kind of vehicle and significancy level

***Variable***	***Kind of vehicle***	***Number(n)***	***Percentage***	***P-value***
Kind of vehicle	Motorcylce	156	39	0.965
	Pedestrian	120	30	
	Car	104	26	
	Other	20	5	
	Total	400	100	
Region of injury	Hand and foot	191	47.7	0.136
	Head	108	27	
	Thorax and abdomen	33	8.3	
	Other	68	17	
	Total	400	100	
Transfer to hospital	Ambulance	340	85	0.821
	Own means	49	12.3	
	Other	11	2.7	
	Total	400	100	

## Discussion

First, all the costs of traffic accidents in Iran are paid by the government and all the health services are provided by public hospitals for the injured person in order to reduce the financial burden of the accident. In this study, among all investigated files, 77.25% of them were for males and 22.75% were for female. Considering the country’s transportation system in which men have a greater share than women, the number of accidents in men was higher than women. In addition, the cost difference between the two groups was significant. The cost in females was 4550.73USD and for males was 10,342.43. In Patel’s study, 92.5% of the participants in the study were male (5), while in another study, 75% of them were male and 25% female (22). The average cost has been reported as 9,580 pounds for men and 10541 pounds for women (23). Because of higher accident costs of men, it may be likely that men drive more recklessly than women, therefore, they have more severe and costly accidents. Considering the cultural conditions of the country, men play a greater and more important role in the national economy, per capita income and gross national product related to the welfare of the community. Damage to this stratum imposes irreparable financial and economic crises to the community and creates unpredictable and excessive social effects.

Age of the patients ranged from one to 94 yr old, and the mean age was 31.5. In Patel’s study, the mean age was reported 36.4; moreover, the highest frequency in the study (48% of the accidents) happened in the age group of 16 to 30 yr (5). About 36% of the accidents were related to the age group of 18 to 29 yr which means that young people were included (22). Damage to this age group not only imposes a cost on the health system (such as medical cost) but also leads to a very high loss of human resources, especially young and active human resources. Therefore, a solution should be provided to prevent the damage. A high number of these events can be attributed to the facts that more people work in this age range and they are not experienced enough and perhaps ignorant of the rules. All of these matters require training for the people, especially the younger age group. Among driving mistakes, ignoring rules was the most important cause of road traffic accident caused due to exceeding speed limit (24). This could be the result of several factors including the young age of the population and urge for driving with a high speed which puts them at risk of accidents (24). In this study, 40.5% of the road traffic accidents have occurred on day and 51.2% at night and 8.3% of them have no reported time. Moreover, about a third of the accidents occur between the hours of 19 and 24. This could be due to poor lighting and darkness of roads and highways. The highest number of the accidents occurred between the hours of 12 and 16; this is a time when people go to their home from work, therefore, there would be a high volume of commuting with vehicles and as a result, there would be more traffic accident during these hours (24). According to the present research, most accidents have been occurred by motorcycle (39%) and there has not been significant difference in cost between different types of vehicles. In Patel’s study, the highest number of traffic-related accidents occurred by car (43.8%) (6); however, in another study, most accidents (73.4%) happened by motorcycles (20). According to Iranian studies, accidents were more common with a motorcycle than a car, which can be due to more carelessness and ignorance of the rules by motorists (25). Based on a study by distal lower extremity is a part of body which most accidents (44%) occurred in them (22). Moreover, the most common RAIs were the upper and lower extremity (57%) (19); in the present study, 47.7% of the RAIs were related to hands and legs, and 27% to head. In terms of anatomical position, hands, feet, and head were the most endangered organs and the possibility of injury to these organs was more than other organs. Due to the sensitivity of head as well as more possibility of giving extensive treatment services in cases of brain and spinal cord injuries, this percent can be considered as a high percent. In some cases, there is more possibility of death and most of the costs are included in this part (26). In our study, 85% of the patients were transferred by ambulance to hospital and there was no significant difference between the hospitals regarding how the patients had been transferred. In another study, 70.83% of the injured persons were transferred by ambulance, 23.61% by the person, and 5.56% by other people (27). As indicated before, the most common way of transferring patients to the hospital is an ambulance. After admission and treatment of the patients in the hospital, 90.8% of the patients were discharged, 5% were discharged against medical advice (DAMA), 1.8% died, and 2.5% were not specified like cases transferred to other centers. The cost difference between the groups was significant (*P*=0.00); this significant difference was due to Death and other outcomes (Table 4). 5.6% of the admitted patients died in the emergency medicine ward and 94.4% of them survived (16). 72.5% of the patients were discharged, 12.7% were discharged as DAMA, 10.2% were admitted to their related ward, and 4.7 % died (28). Statistically, Tuckey test revealed a significant difference between death and other outcomes. It can be due to more intensive care, and even longer, such as the operating room, anesthetic, ICU, and so on. The results of other studies showed a number of risk factors from traffic accidents including being a male, having good vehicles, unsafe road conditions, high traffic volume, low light at night, and driving during weekends. These risk factors not only increased the possibility of loss of life but they are also considered as higher risk for the severity of traffic accidents. Moreover, individuals with less than two years of history of driving were considered to have a higher risk of traffic loss (29). Additionally, level of education can be effective in the number of traffic accidents. Higher level of education may lead to lower possibility of traffic accidents (24).

Direct costs in healthcare have been estimated to be 80 million dollars (522 euro per person) and about 18% of the costs were due to rehabilitation. More costs for traffic accident allocated to men, older patients, severer injuries (3075 €), blood vessels injuries (2688), and brain trauma injuries (30). These results were consistent with our results because, in the first place, our study showed that the costs of men were higher than women; secondly, in terms of costs, patients with death outcome had a significant difference with other outcomes in which the death occurred because of severe injuries. Finally, the present study showed that there was a significant difference in the costs of patients in the ages of 46 to 60 and the other age groups. There is more need for longtime care in this range of age, these results could be assumed logical.

## Conclusion

Traffic accidents have high financial burden in GDP in Iran. More than one-fourth of the traffic accidents have occurred in head and the highest frequency of accidents have been among motorcyclists. Given the necessity of using helmets for riding motorcycles, current rules had not been effective enough. Therefore, more effective rules should be enacted with regard to use of helmets for motorcycling. On the other hand, since about half of the traffic accidents were related to the age of 16 to 30 who were the active population of a community, therefore, any disability or death in these groups could cause an economic burden on the community and increase DALY. Thus, it appears essential to develop proper programs such as education for appropriate driving and strict rules for giving driving license to these ages.

## Ethical considerations

Ethical issues (Including plagiarism, informed consent, misconduct, data fabrication and/or falsification, double publication and/or submission, redundancy, etc.) have been completely observed by the authors.
